# Barriers to the implementation of large-scale electronic health record systems in primary healthcare centers: a mixed-methods study in Saudi Arabia

**DOI:** 10.3389/fmed.2025.1516714

**Published:** 2025-04-15

**Authors:** Haitham Alzghaibi, Hayley A. Hutchings

**Affiliations:** ^1^Department of Health Informatics, College of Applied Medical Sciences, Qassim University, Buraydah, Saudi Arabia; ^2^Swansea University, Medical School, Swansea, United Kingdom

**Keywords:** electronic health records, barriers, primary healthcare centers, large-scale IT projects, Saudi Arabia, questionnaire, semi-structured interviews, mixed methods

## Abstract

**Background:**

In the past two decades, policymakers have increasingly prioritized the integration of technology to enhance healthcare quality and efficiency. However, nearly half of these initiatives have failed to achieve their intended objectives due to various challenges, including financial constraints and implementation complexities. The Saudi Ministry of Health (MoH) launched a nationwide initiative to implement an Electronic Health Record System (EHRS) across approximately 2,200 Primary Healthcare Centers (PHCs). However, previous attempts at deployment encountered significant obstacles, leading to project failure. Key challenges identified by the MoH included inadequate infrastructure, limited connectivity, and lack of system interoperability.

**Aim:**

To explore the key barriers hindering the effective implementation of EHRS in PHCs in Saudi Arabia, with a focus on technical, organizational, and user-related challenges.

**Method:**

This study adopted a mixed methods approach using an exploratory sequential design to capture both strategic and operational perspectives on EHRS implementation. The qualitative phase involved semi-structured interviews with 14 key informants from the MoH who were directly involved in the EHRS deployment, aiming to identify structural and policy-related barriers. The quantitative phase consisted of an online survey completed by 351 PHC practitioners to assess user-level challenges, including system usability, training adequacy, and technical support availability. This sequential approach ensured that the survey was informed by the insights gained from the qualitative phase.

**Results:**

Findings from both phases revealed multiple barriers affecting EHRS implementation. Key challenges included the large-scale nature of the project, resistance to change, insufficient training, lack of technical support, poor system interoperability, geographical limitations, and inadequate user engagement. Additionally, unclear software selection criteria contributed to integration difficulties. To address these barriers, the study proposes several strategies, including collaborating with telecom providers to improve connectivity, implementing a phased regional deployment strategy, and enhancing training and technical support frameworks.

**Conclusion:**

The study highlights insufficient connectivity, inadequate technical support, and high turnover in key leadership positions as major contributors to previous implementation failures. Notably, training and ongoing support emerged as critical obstacles, whereas concerns related to privacy and confidentiality were found to be less significant. To ensure successful EHRS adoption, decision-makers must allocate sufficient resources for software selection, infrastructure improvements, workforce training, and continuous technical support. This study fills a research gap by providing evidence-based recommendations for optimizing large-scale EHRS implementation in healthcare settings, particularly in resource-constrained environments.

## Introduction

The implementation of Electronic Health Record Systems (EHRS) has become a fundamental aspect of modern healthcare, with widespread adoption driven by national policies and international health IT regulations. Many countries, including those in Europe, have established guidelines to ensure standardized EHRS integration, data security, and interoperability within healthcare systems (1–5). Similarly, Saudi Arabia’s Vision 2030 prioritizes digital transformation in healthcare, with initiatives such as the Saudi Health Information Exchange (SHIE) and the National eHealth Strategy, which focus on improving data interoperability and ensuring compliance with international health IT standards (5, 6). However, despite these regulatory efforts, EHRS implementation remains a complex and often challenging process, particularly in regions where infrastructure, technical expertise, and institutional readiness vary significantly. Studies indicate that failure rates for EHRS adoption range between 50 and 70% across different healthcare settings, highlighting persistent barriers related to financial constraints, resistance to change, and usability concerns (7–14). Understanding these challenges is crucial for developing targeted strategies to facilitate successful EHRS deployment, particularly in contexts where healthcare digitisation is still evolving.

A growing body of research highlights multiple challenges hindering the successful deployment of EHRS. These include technical limitations, financial constraints, usability issues, interoperability challenges, and resistance from end users (15–17). In developing countries, additional systemic and structural barriers further impede EHRS adoption. Many low- and middle-income countries (LMICs) struggle with unreliable internet connectivity, outdated computer systems, and insufficient national policies supporting health information technology (14, 18–20). Healthcare facilities in these regions often lack the financial resources for large-scale digital transformation, continuing to rely on paper-based record-keeping systems, which makes the transition to EHRS both logistically challenging and cost-intensive (14, 20, 21).

Several case studies illustrate these challenges. In Uganda, insufficient government funding and inadequate staff training significantly hindered the effective use of EHRS, resulting in low adoption rates and underutilization of installed systems (22). Similarly, in Kenya, although national efforts have been made to implement EHRS in public hospitals, challenges such as poor internet connectivity, frequent power outages, and limited digital literacy among healthcare workers have significantly slowed progress (23). In India, while some urban hospitals have successfully integrated EHRS, many rural healthcare centers continue to rely on manual record-keeping due to budget constraints, resistance from older healthcare professionals, and a lack of standardized national policies (24). In United Arab Emirates, where the government introduced a national EHRS initiative, implementation has been uneven, with major urban centers adopting the system more effectively than smaller municipalities, primarily due to regional disparities in IT infrastructure and funding allocation (25). These examples highlight the ongoing challenges in developing nations, where even when EHRS are introduced, they often fail to achieve full adoption due to structural and economic barriers.

Among these barriers, system usability and interoperability failures have emerged as critical issues affecting EHRS adoption. Poor usability has been directly linked to low adoption rates, user dissatisfaction, and reduced clinical efficiency (15, 26–28). Conversely, research suggests that enhancing EHRS usability can improve acceptance and long-term adoption among healthcare professionals (5, 11, 27). Additionally, interoperability failures, often resulting from incompatible systems and the absence of standardized communication protocols, prevent seamless data exchange across healthcare institutions, further limiting the system’s effectiveness (1, 3, 11, 15, 29).

Despite extensive research on EHRS implementation, there remains a critical gap in understanding the unique challenges faced by PHCs, particularly in Saudi Arabia. While existing studies have explored general barriers to digital health adoption, limited research has examined the contextual factors affecting EHRS implementation in PHCs within the region. Given the Saudi Ministry of Health’s (MoH) ongoing efforts to implement EHRS nationwide, addressing these barriers is essential to ensuring the successful deployment, sustainability, and effectiveness of the system.

Study aim: To explore the key barriers hindering the effective implementation of EHRS in PHCs in Saudi Arabia, with a focus on technical, organizational, and user-related challenges.

- To examine the technical, organizational, and user-related barriers to EHR implementation in PHCs in SA from the perspectives of project team members and end users.
- To assess the perceptions and experiences of end users regarding EHR usability, data management, and considering their IT skills and familiarity with digital health technologies.
- To investigate the challenges faced by project team members in deploying EHR systems, including infrastructure limitations, system interoperability, and administrative constraints.
- To differentiate between barriers related to personal health data management and clinical data documentation within the EHR system, identifying specific challenges based on users’ roles.
- To provide evidence-based recommendations for improving EHR implementation and adoption in PHCs.

What this study adds:

- This study provides practical insights into the real-world procedures and steps involved in implementing EHRS, offering a framework that can guide healthcare institutions in overcoming deployment challenges.
- By identifying and analyzing key barriers to EHRS adoption, the findings contribute to a deeper understanding of how to mitigate organizational, technical, and policy-related impediments, ultimately supporting the successful implementation of EHRS in diverse healthcare settings.
- This study addresses critical research gaps by focusing on primary healthcare centers (PHCs) in Saudi Arabia and other Arab Gulf Countries (GCCs), where EHRS implementation faces unique infrastructural and regulatory constraints.
- Methodologically, this study advances EHRS research by employing a mixed-methods approach, integrating qualitative and quantitative analyses to provide a comprehensive, evidence-based examination of the obstacles encountered in real-world EHRS deployment.

## Methods

### Research design and data collection

This study employed a mixed methods approach using an exploratory sequential design to examine the barriers to EHR implementation in PHCs in Saudi Arabia. This approach was selected to ensure a comprehensive understanding of the challenges from both strategic and operational perspectives. By integrating qualitative and quantitative data collection, the study aimed to first explore key issues through in-depth discussions with policymakers and project team members before validating these findings through a structured survey of end users. This sequential design allowed for a more targeted investigation, ensuring that the survey captured the most relevant and context-specific barriers identified in the qualitative phase.

The qualitative phase involved semi-structured interviews with project team members and policymakers who were responsible for the planning, development, and implementation of the EHR system. These interviews sought to uncover technical, organizational, and policy-related barriers, including infrastructure limitations, system interoperability challenges, administrative constraints, and strategic decision-making processes. Additionally, particular attention was given to the distinction between barriers related to personal health data management and clinical documentation, as these aspects influence compliance, usability, and overall adoption of the system. The insights gained from this phase informed the development of the subsequent survey by ensuring that it addressed the most pressing concerns identified by key decision-makers.

The quantitative phase consisted of a close-ended survey administered to end users of the EHR system, specifically healthcare professionals working in PHCs. This phase aimed to measure the prevalence and significance of the identified barriers from the perspective of those directly interacting with the system in their daily clinical practice. The survey assessed usability challenges, data management issues, and the role of IT skills in effective system utilization. By focusing on end users’ experiences, the study provided empirical evidence on how implementation barriers translate into real-world difficulties, affecting workflow efficiency, data accuracy, and overall system adoption.

To ensure methodological rigor and validity, both the semi-structured interview guide and the survey instrument were adapted from previously validated tools developed by Alzghaibi and Hutchings (5). This iterative and structured approach enabled the study to first identify key barriers qualitatively and then validate and quantify their impact through quantitative analysis. By integrating the perspectives of both policymakers and end users, the study offers a holistic, evidence-based understanding of the challenges hindering EHR adoption in PHCs. This approach also ensures that the study’s findings are directly applicable to policy recommendations, system design improvements, and strategies for enhancing user engagement and system usability.

### Study questionnaire validity and radiality

The authors performed multiple initial procedures to evaluate and, if needed, enhance the questionnaire. The questionnaire was developed in two phases. The initial instrument underwent evaluation by a panel comprising external specialists, including: two information technology experts from the head quarter of the Saudi MoH, the directors of the information technology departments of two distinct hospitals, one scholar from King Saudi University (holding a doctorate in Health Informatics), one radiologist, and one pharmacist (holding a master’s degree in Health Informatics).

After undergoing a review and receiving input from expert panels, the questionnaire was administered as a pilot study to a small group (n = 5) of the project team. This was done to verify the clarity, comprehensibility, and reliability of the questionnaire. The data collection questionnaire was tested at the Saudi Ministry of Health’s main office with project team members who willingly consented to participate. Various factors were considered, including distinct position tiers (e.g., supervisors, directors, and senior managers), diverse divisions, and varied ethnicities.

Table 1 demonstrates that the dependability of all scales is satisfactory. The obstacles scale exhibits the highest level of dependability, with a coefficient of 0.83, followed by negative attitudes with a coefficient of 0.75. The overall reliability of the data collection device in the study is high, with a coefficient of 0.81.

**Table 1:** Cronbach’s alpha test.

Scale	Number of items	Cronbach’s alpha
Negative attitude	7	0.75
Barriers	16	0.83
Entire questionnaire	23	0.81

### Population and sampling

A non-probability, purposive, snowball sampling approach was employed for the qualitative phase of this study to ensure the selection of participants with direct experience in EHRS deployment. This method allowed for the identification of key individuals actively involved in the implementation process within PHCs in Saudi Arabia, ensuring that insights were gathered from those with firsthand knowledge of the system’s challenges and facilitators.

A total of 53 project team members from the Saudi Ministry of Health (MoH) headquarters were invited to participate in semi-structured interviews, as they played a central role in EHRS implementation. These participants held various positions, including general managers, directors, and technicians, representing both strategic decision-making and operational roles. Their diverse professional backgrounds, spanning information technology (IT) and clinical fields, provided a comprehensive perspective on the barriers and enablers of EHRS adoption.

Ultimately, 14 participants were interviewed, as this number was deemed sufficient to achieve data saturation, where no new themes or insights emerged in the final interviews. The informative nature of the discussions compensated for the sample size, with some interviews lasting up to 2 h, allowing for an in-depth exploration of implementation challenges, system usability, technical limitations, and organizational factors. Additionally, pragmatic considerations such as participant availability and the necessity of conducting detailed, time-intensive interviews influenced the final sample size. The selection of 14 participants was driven by informational sufficiency and thematic saturation, rather than a predefined numerical target, aligning with best practices in qualitative research. A detailed analysis of participant characteristics will be presented in the results section.

For the quantitative phase, a multi-stage cluster sampling technique was employed to ensure a geographically representative sample of healthcare professionals involved in EHRS implementation across PHCs in Saudi Arabia. This method was particularly beneficial for conducting large-scale studies and addressing the challenges associated with random sampling in a widely dispersed population.

- Stage one: The Saudi Ministry of Health’s regional classification system was used to divide the country into five geographical clusters.
- Stage two: Within each cluster, simple random sampling was applied to select specific provinces, ensuring balanced representation across different geographic regions.
- Stage three: A total of 21 PHCs were randomly selected from the 2,259 PHCs within the five designated clusters see Table 2.

**Table 2:** Geographical distribution of selected PHCs with EHRS implementation in Saudi Arabia.

No.	Region	Geographical location	Number of PHCs with EHRS	Selected in this study	Number of selected PHCs
1	Riyadh	East	21	Yes	5
2	Gassim	Centre	12	Yes	4
3	Makkah	West	16	Yes	4
4	Almadinah	West	11	No	
5	Alsharqiah	East	10	No	
6	Albaha	South	9	Yes	4
7	Asir	South	13	No	
8	Najran	South	11	No	
9	Hail	North	9	No	
10	Alshamaliyah	North	11	No	
11	Jazan	South	10	No	
12	Tabuk	North	9	No	
13	Aljouf	North	8	Yes	4
Total	13		150		21

The target sample size of 491 participants was determined based on the total population of 38,514 healthcare professionals working in PHCs across Saudi Arabia. Standard sampling formulas were used to achieve a representative sample, ensuring an acceptable margin of error and confidence level for the study. The sample was proportionally distributed across the selected centers to capture diverse perspectives from healthcare professionals in different regions. Ultimately, 351 completed responses were received, resulting in a response rate of approximately 71.5%, which is considered statistically robust for survey-based research in healthcare settings. The multi-stage cluster sampling approach proved to be an effective strategy for obtaining a diverse and representative sample, enabling a comprehensive assessment of the barriers affecting EHRS implementation in PHCs.

### Data collection process

#### Qualitative data collection

Fourteen semi-structured interviews were conducted over 2 months with key informants directly involved in EHRS implementation. These participants were strategically selected from the Saudi MoH headquarters, representing various levels of decision-making and operational roles. Each interview was scheduled for a maximum of 2 h, though participants had the flexibility to extend or conclude the session as needed. To ensure convenience and minimize disruptions, interviews were conducted at a consistent location, except for one, where the researcher traveled to the participant’s workplace. A structured interview guide was developed to maintain rigor and consistency, while allowing flexibility for follow-up questions. Before each interview, participants were provided with detailed study information, given adequate time to review the informed consent form, and provided explicit verbal confirmation of their willingness to participate. Ethical approval for data collection was obtained in advance.

Interviews were digitally recorded using secure devices, ensuring data reliability. To enhance security, recording devices were placed on airplane mode, fully charged, and had sufficient storage capacity. Field notes were taken to document nonverbal cues and emerging themes, allowing for real-time refinement of questions. This approach ensured a comprehensive and nuanced understanding of the barriers to EHRS implementation. The interviews lasted between 35 and 130 min, with an average duration of approximately 65 min. Data saturation was achieved, as no new themes emerged in the final interviews, confirming the sufficiency of the sample size.

#### Quantitative data collection

To maximize response rates and ensure efficient data collection, an online self-administered questionnaire was disseminated via SurveyMonkey. This approach was selected due to Saudi Arabia’s vast geography, limited postal infrastructure, and the study’s large sample size, making physical distribution impractical. The questionnaire was distributed over 10 weeks, with two reminder emails sent in the second and fourth weeks to encourage participation. Since official PHC staff emails were not widely used, alternative methods were employed to ensure accessibility and broad participation. A direct communication strategy was implemented, involving 21 designated PHC representatives. Each representative received a copy of the ethical approval letter and was invited to join a dedicated WhatsApp group created for the study. All 21 representatives agreed to participate, facilitating an efficient and adaptive dissemination process. A single survey link was shared via WhatsApp, with instructions to complete the questionnaire and distribute it among colleagues using personal email and other communication channels. This multi-channel dissemination strategy helped overcome logistical challenges and ensured wide participation, reinforcing the importance of flexible approaches in large-scale healthcare research.

### Data analysis

#### Qualitative

Thematic analysis was used to analyze the qualitative data obtained from the semi-structured interviews (30). Thematic analysis was chosen due to its versatility and lack of limitations to a particular framework or theory. Furthermore, the choice was taken to do theme analysis to discern patterns in highly abundant information from various viewpoints (30). After concluding the interviews, the authors transcribed the audio recording into a textual format and subsequently saved it as a Microsoft Word document. The recordings were transcribed word for word to ensure the response context and information substance are easily understood. Hence, the data analysis commenced promptly after the transcription of the interviews, followed by their translation into English (for interviews done in Arabic). The transcripts were Microsoft Word files that were put into the NVivo program.

#### Quantitative

The statistical analysis was conducted using SPSS Version 27. To ensure the reliability and internal consistency of the study’s measurement scales, Cronbach’s alpha was computed. Following this, descriptive statistics were employed to summarize the data collected from participants, including median values, percentages, total agreement scores, and ranking of items. The total agreement percentage was calculated by summing the number of respondents who selected “agree” or “strongly agree,” allowing for a structured comparison of items within each scale. These scores were then ranked in descending order, highlighting the most strongly endorsed aspects of the EHRS implementation experience. However, it is important to note that the barriers scale did not include total agreement measurement, as it was analyzed separately based on response distribution.

After presenting the descriptive statistics, inferential statistical tests were conducted to examine correlations and differences between groups. Given the ordinal and nominal nature of the data, only non-parametric tests were used. Specifically, Mann–Whitney U tests were applied for comparisons between two groups, while Kruskal-Wallis tests were employed for comparisons involving three or more groups. These tests were chosen to account for non-normal data distribution and the categorical structure of responses (31).

To enhance data visualization and facilitate interpretation, R software was used to generate graphs and visual representations of key findings. This approach provided a clearer depiction of trends, group differences, and the overall distribution of responses, supporting a more comprehensive analysis of EHRS implementation barriers and facilitators.

## Results

Fourteen individuals from the Saudi Ministry of Health’s project team who were instrumental in implementing electronic health records in primary healthcare facilities consented to an in-person interview. While a total of 351 professionals from 21 Primary Health Centers (PHCs) took part in the online survey. The study findings were previously published in a Ph.D thesis of the main author (32).

### Interview results

The participants held various positions, including three General Managers (GM), three Heads of Department (GM), one Deputy Head of Department (DHD), one Software Developer (SD), and five Data Analysts (DA).

This section encompasses all the themes and codes that have been established to signify the obstacles to the installation of EHRS in PHC facilities in Saudi Arabia. The participants identified multiple obstacles. In addition, many measures to overcome the obstacles that can result in the failure of implementation were documented. At first, the participants acknowledged that there are multiple obstacles to establishing EHRS in PHCs.

*“The obstacles are too many.”* (SD1).

During the interviews, the participants cited numerous challenges they faced while participating in the deployment of EHRS in PHCs. The barriers to EHRS implementation encompassed various factors, including personnel turnover, a dearth of expertise in EHRS implementation, inadequate training, a high number of primary healthcare centers, limited implementation time, challenges associated with vendors, insufficient connectivity, subpar infrastructure, geographical obstacles, EHRS interoperability, software selection, and a shortage of suitable computers (see Table 3) (31).

**Table 3:** Themes and sub-themes.

No. of themes	Themes	No of sub-themes	Sub-themes
1.	Human barriers	1.	Changing people
		2.	Shortage of expertise in EHRS implementation
		3.	Lack of training
2.	Logistical barriers	4.	Scale of the project
		5.	Timeframe for implementing EHRS in PHCs
		6.	Geographical challenges
3.	Technologically barriers	7.	Inadequate infrastructure and lack of connectivity
		8.	Software selection
		9.	Lack of EHRS interoperability
4.	Overcoming barriers	10.	Continuous development
		11.	Compartmentalization
		12.	Piloting the system
		13.	Cooperation with TelCo

#### Theme one: human barriers

##### Changing people

Projects to deploy EHRS are negatively affected by personnel changes, particularly those involving politicians or top administrators. Every incoming minister brings a fresh plan that completely eradicates all past efforts, including both planned and implemented strategies. Modifications extended beyond plans and tactics, encompassing the alteration of administrative personnel and decision-makers.

*“Every minister cancels the previous plan and develops new plans and strategies.” (GM 2).*

*“Unfortunately, we have a problem in SA in that new ministers remove the plans and decisions of the former minister.”* (DHD 1).

As a result of these modifications, obstacles and interruptions have negatively impacted the progress of numerous EHRS installation initiatives.

*“Some obstructions, delays and execution of works are also affected by constant changes that happen in the Ministry, including replacement of the Minister and some seniors in the MoH.”* (GM 1).

*“Unfortunately, frequent changes at the ministerial level and the subsequent changes at the departmental level had a negative effect on the completion of the projects.”* (GM 2).

The ramifications of these alterations encompass not only the prolongation and disturbances to the projects, but also the cessation of ongoing projects (such as the adoption of EHRS in Primary Health Centers in Saudi Arabia).

*“Unfortunately, with new seniors and a new Minister, the work on the current project stopped.”* (HD 3).

Likewise, alterations in the personnel composition of Primary Health Centers (PHCs) were discovered to have an adverse effect on the adoption of Electronic Health Record Systems (EHRS), specifically in regards to the delivery of training.

*“Another problem that has been encountered is that of staff change. Many times, after three or 4 months of training one employee, another employee comes instead of that one.”* (SD1).

##### Shortage of expertise in EHRS implementation

The Ministry of Health in Saudi Arabia faces a deficiency of skilled personnel, including IT technologists and health informatics professionals.

*“One of biggest challenges is finding talented people in specific areas, especially in SA.”* (GM1).

*“Finding efficiencies is a very difficult task; we also have a very big problem with labor availability.”* (HD1).

##### Lack of training

The Saudi Ministry of Health (MoH) encounters the additional obstacle of training, specifically in relation to the utilization and execution of Electronic Health Record Systems (EHRS) at Primary Health Care Centers (PHCs). A significant proportion of the survey participants concurred that training posed a substantial obstacle to the implementation of Electronic Health Record Systems (EHRS). As an illustration:

*“The lack of training is among the problems.”* (HD 1).

*“Of the main obstacles is training.” (Analyst 1).*

#### Theme two: logistical issue

##### Scale of the project

The abundance of Primary Health Centers (PHCs) is a significant hindrance to the successful implementation of Electronic Health Record Systems (EHRS). The magnitude of the project has a detrimental impact on the provision of training and technical assistance.

*“The biggest problem is how to install the EHRS into more than 2,000 PHCs, so it is a huge issue because it is equally important as the hospitals.”* (Analyst 3).

*“The main obstacles are training and technical support in particular; with regards to the PHCs, problems arise due to the large number of the PHCs.”* (Analyst 1).

##### Timeframe for implementing EHRS in PHCs

The project team encountered the difficulty of time constraints when implementing a large-scale project, as such projects necessitate a greater amount of time and have the potential to result in delays.

*“Our problems are often associated with time, as the implementation of such large projects requires a lot of time.”* (HD3).

##### Geographical challenges

The Saudi Ministry of Health considers the size and geographical characteristics of Saudi Arabia to be a significant problem.

*“The geographical nature of the Kingdom is considered to be a big challenge to EHRS implementation.”* (HD 3).

The geographical distribution of certain PHCs poses a significant challenge to the introduction of EHRS across the Kingdom. Especially those situated in rural or isolated regions.

*“The most influential obstacles to the MoH are PHCs which are located in remote areas.”* (GM 3).

#### Theme three: technologically barrier

##### Inadequate infrastructure and lack of connectivity

The aforementioned geographical challenges are directly correlated with infrastructure. Therefore, the establishment of infrastructure and the establishment of connectivity are two significant obstacles to the introduction of EHRS in PHCs in Saudi Arabia. The Saudi MoH considers the issue of connectivity between PHCs to be a challenging problem, which has resulted in the postponement of numerous projects. The absence of connectivity among PHCs is a consequence of inadequate infrastructure.

*“Also, we faced other difficulties related to the infrastructure such as connectivity, especially with PHCs in remote areas.”* (GM 1).

*“The technical side is a very important factor regarding the development of the infrastructure and networks, it is possible to choose the best system in the world, but when it comes to implementation, the surprise is that the infrastructure may not be suitable for implementation.”* (Analyst 1).

Establishing a suitable infrastructure might incur significant costs, particularly due to the utilization of rented facilities for certain PHCs that are not conducive to IT initiatives.

*“Infrastructure involves a very expensive process of communication between the health centers, especially since some of the PHCs are in rented buildings.”* (GM 3).

Another infrastructure-related obstacle is that not all PHCs have computers and other necessary devices.

*“Lack of computers is one of the obstacles we faced at the beginning, especially in non-developed PHCs.”* (Analyst 2).

##### Software selection

Another obstacle to implementing EHRS in Saudi PHCs is the absence of a suitable EHRS that aligns with the project team’s goals and aspirations. Most of the EHRS offered to the Saudi MoH, especially those from outside the country, do not align with the features and operations of primary healthcare facilities in SA.

*“We also face a big challenge, we cannot find either a local or global EHRS that meets the requirements of PHCs in SA, and the most important reason is that there are not many options for PHCs because all global companies focus on hospitals and making systems that fit into hospitals.”* (HD 3).

The characteristics of PHC facilities in Saudi Arabia differ from those in other nations including the United Kingdom, United States, and Australia. The disparities have impeded the process of choosing software because there is a scarcity of a supremely effective and internationally renowned EHRS that aligns with the existing functionalities of PHCs in SA.

*“…and what made it even more difficult is the differences in the characteristics of PHCs and business workflow in SA compared to that of large countries such as America, Britain and Australia. They apply so-called ‘GPs’ rather than PHCs.”* (HD 3).

*“We did not find an EHRS that is compatible with the workflow of the PHCs in SA.”* (GM 3).

Vendor selection is a hindrance to the implementation of EHRS due to many factors, such as suppliers’ inefficiency, price escalation, and lack of adequate experience in the Saudi healthcare system. These three criteria hinder the identification of appropriate vendors for EHRS installation initiatives. The project team hesitated to make a conclusion in this area due to a deficiency in experience. In addition, inflated costs posed a challenge for the Saudi MoH in pursuing an arrangement with vendors. Some vendors could not meet the project team’s ambitions at the MoH, as evidenced by the quotations below:

*“International companies have never implemented EHRS Saudi PHCs; this has made us hesitant to select international companies.”* (GM 3).

*“The main obstacles are contracting with a qualified vendor.”* (SD 1).

##### Lack of EHRS interoperability

The lack of interoperability in EHRS has been identified as a significant obstacle to the successful adoption of EHRS programs in primary healthcare centers in SA.

*“One of the big challenges here is EHRS interoperability.”* (Analyst 3).

*“We should take EHRS interoperability issues seriously.”* (SD 1).

#### Theme four: overcoming barriers

The Saudi MoH has implemented several measures to overcome the aforementioned obstacles and improve the effectiveness of EHRS implementation. Measures taken to address the aforementioned barriers and difficulties involve establishing the essential infrastructure and protocols, carrying out extensive research and studies, collaborating with Telecommunication Companies (TCs), engaging multiple vendors, dividing the Kingdom into distinct regions, and actively involving all relevant stakeholders.

##### Continuous development

The Saudi MoH successfully addressed difficulties and problems in implementing EHRS by focusing on developing the existing infrastructure, standards, and other technical factors.

*“The key success, of course, is the development of standards and infrastructure.”* (Analyst 3).

The MoH also intends to enhance the current EHRS to align with the goals of the MoH and meet the needs of its users.

*“The Ministry is currently studying the possibility of the development of the previous EHRS to be generalized and implemented in all PHCs.”* (HD 1).

#### Compartmentalization

Due to its extensive scope, the implementation of the EHRS in PHCs is highly complex. To address this challenge, the MoH has implemented a strategy to divide the country into five zones. As part of this strategy, a data center will be established in each of these regions. Through these data centers, all PHCs and hospitals within each region will be interconnected.

*“As I mentioned to you earlier, the plan was to set up a data center in each region after the division of the Kingdom into five regions (zones), to link the PHCs with these data centers, and then connect the PHCs with the hospitals.”* (HD 3).

*“We divided the Kingdom into five zones; each zone will have one data center.”* (GM 1).

Multiple vendors will be chosen to implement the EHRS in order to decrease the burden and minimize the risks.

*“We will be contracting with at least three providers to reduces the pressure on the provider. If it is one vendor, the number to cope with is huge, and one company alone cannot implement the EHRS in all PHCs in SA.”* (GM 1).

##### Piloting the system

The Saudi MoH will conduct a trial of the chosen system by introducing the EHRS in a limited number of PHCs. Subsequently, the MoH will assess the system to identify any potential usability or technical difficulties before proceeding with the real installation.

*“First, we will select a system and try to implement it in some PHCs for the evaluation of several aspects to measure the system’s usability and determine any problems; then we will collect and analyze the problems and solve them. We will work on this more than once until we achieve 100% user satisfaction.”* (GM 3).

#### Cooperation with TelCo

Collaborating with communications and information technology firms is another potential strategy that could help overcome the constraints. The Saudi MoH has entered into agreements with multiple communication and IT firms. The objective of this collaboration is to address the geographical issues and accompanying obstacles related to infrastructure.

*“The geographical challenges will be addressed through co-ordination with the TCs and connection to the Internet for all PHCs; then linking them to the data centers in each region.”* (HD 1).

*“Working in co-ordination with the TCs on the development of infrastructure.”* (Analyst 1).

### Questionnaire results

Out of the 491 healthcare practitioners working in the selected PHCs, 351 completed in the questionnaire. This equated to a response rate of 71.5%. Therefore, the questionnaire data were collected from 351 participants across five different regions of the Kingdom of Saudi Arabia. Out of all the respondents, the highest number, 103 (29.3%), lived in the capital city, Riyadh (refer to Table 4).

**Table 4:** Association between demographic variables and attitudes toward EHRS and perceived barriers: results from Kruskal-Wallis and Mann–Whitney U tests.

Demographic variables	Test used	Negative attitude (*p*-value)	Level of barriers (*p*-value)
Occupation	Kruskal-Wallis Test	0.166	0.964
Regions	Kruskal-Wallis Test	0.610	0.221
Experience in position	Kruskal-Wallis Test	0.765	0.833
Experience with using an EHRS	Kruskal-Wallis Test	0.303	0.570
Experience in using a personal computer	Kruskal-Wallis Test	0.804	0.218
Age	Kruskal-Wallis Test	0.379	0.542
Gender	Mann–Whitney U Test	0.113	0.338

All participants were employed in healthcare and administrative positions. Table 4, displays the distribution of job roles among the participants. Out of the total, 149 individuals (42.4%) held administrative positions, such as managers, secretaries, and receptionists. 104 participants (29.6%) worked in nursing roles, while 32 (9.1%) were physicians and 30 (8.5%) were pharmacists. Four individuals, accounting for 1.1% of the total, did not disclose their occupation.

Age was quantified using six distinct categories, as depicted in Table 4. Out of the total participants, 192 individuals, accounting for 54.7% of the sample, fell between the age range of 25–34 years. Table 4 provides a comprehensive analysis of the age groups. Four individuals, accounting for 1.1% of the total, did not disclose their age. Participants were additionally requested to indicate their gender. The majority of participants were male, with a total of 261 individuals, accounting for 74.4% of the sample. Among the 351 participants, the number of females was only 81, accounting for 23.1% of the total. Nine individuals, accounting for 2.6% of the total, did not disclose their gender.

The participants’ usage of a personal computer at home exhibited variation, with the majority of participants (36.8%) reporting experience spanning from 10–15 years. Table 5 shows that a mere 18 participants, accounting for only 5.1% of the total, had less than 1 year of experience using a personal computer. Four participants, accounting for 1.1% of the total, did not disclose their level of experience in using a personal computer at home.

**Table 5:** Spearman’s correlation coefficient for correlations between barriers and negative attitude.

		Barriers	Negative attitude
Barriers	Correlation Coefficient	1.000	−0.176^*^
	Sig. (2-tailed)	.	0.012
Negative Attitude	Correlation Coefficient	−0.176^*^	1.000
	Sig. (2-tailed)	0.012	.

*Weak correlation.

**Figure 1:**
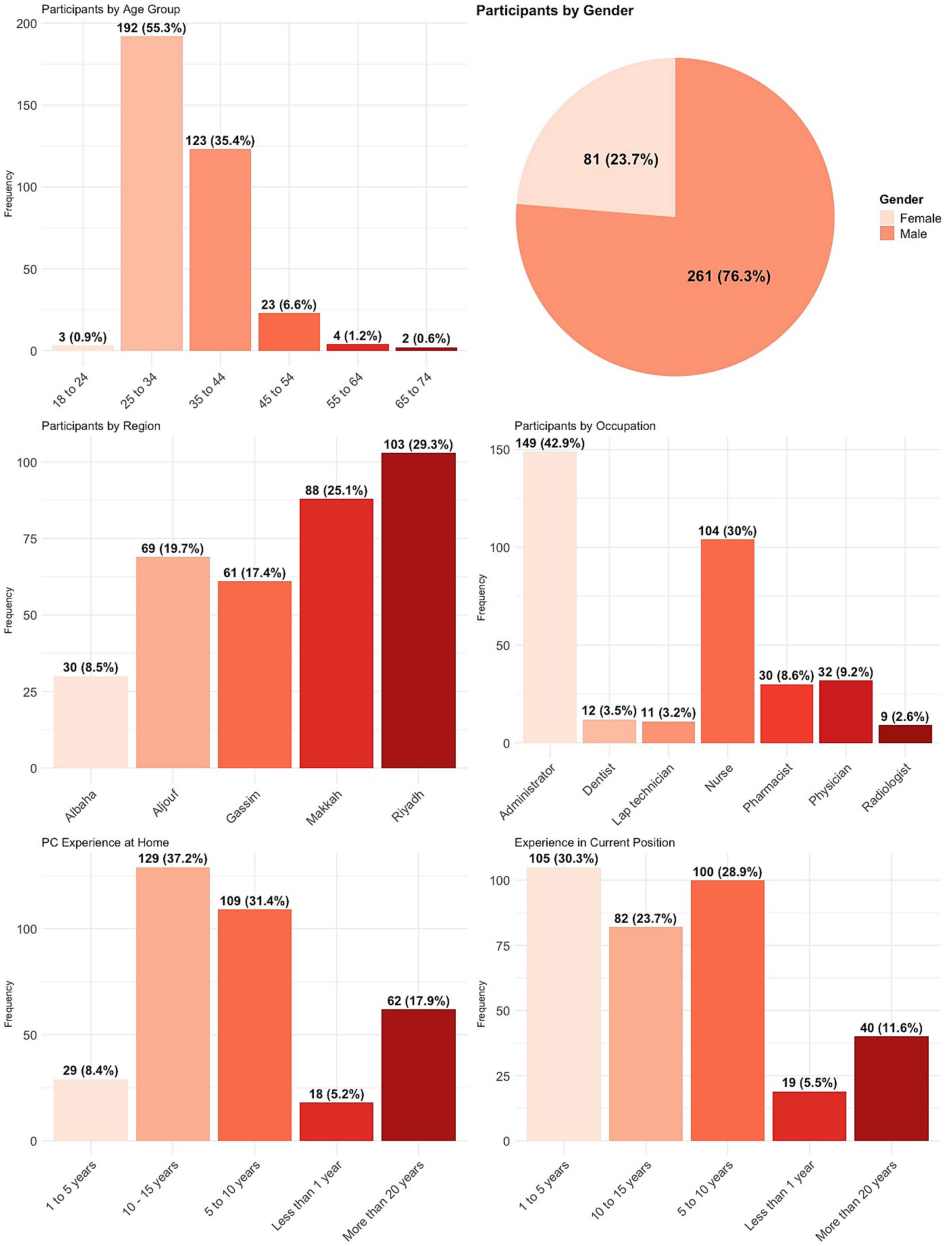
Participant demographic distribution.

The duration of the participants’ engagement in their present work position was assessed using five distinct categories. Out of all the participants, a majority of 105 individuals, accounting for 29.9% of the total, had a professional experience ranging from one to 5 years. Table 4 provides a comprehensive analysis of the amount of time participants have spent in their current position. Five participants, or 1.4% of the total, did not disclose their experience in current position (31) (Figure 1).

#### Negative attitudes toward the EHRS

**Figure 2:**
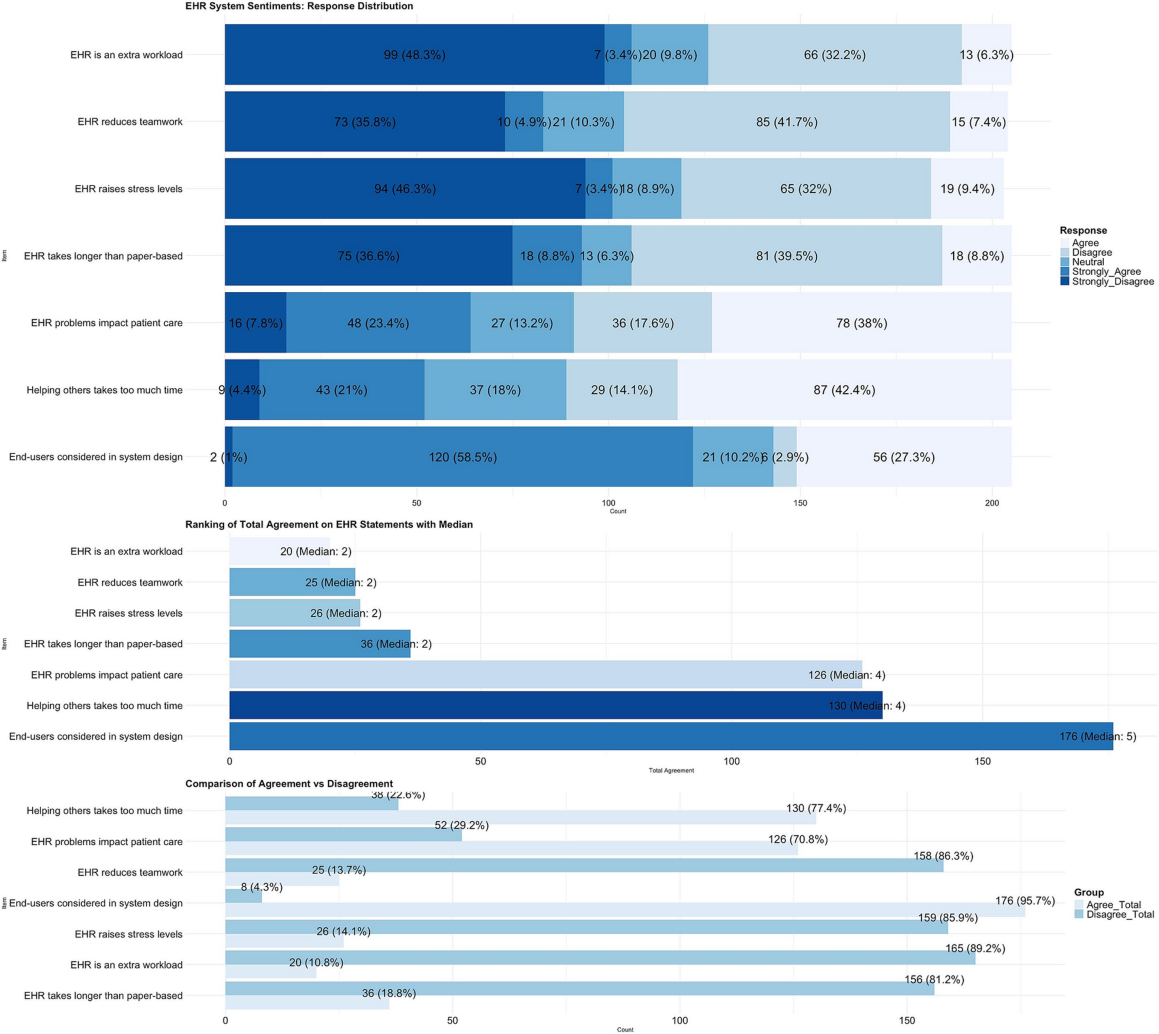
Participant responses to seven items reflecting negative attitudes toward the EHRS implemented in PHCs.

Figure 2 includes elements that indicate several characteristics that impact the deployment of EHRS, such as user engagement and system effectiveness. These factors were determined based on the replies to seven questions on the negative attitude scale. The negative attitude scale collects participant responses to items that describe issues affecting the implementation of EHRS, such as the involvement of EHRS end-users. Consequently, the utmost level of support was generated for user engagement: (1) “*End-users should have been considered in the system design*” (85.8%). The second level of agreement was generated for (2) “*It takes too much time to help others who do not know how to use the system”* (63.4%). However, lower agreement was generated for items: (5) “*Using EHRS raises stress levels among practitioners*” (12.8%); (6) “*The system makes me feel like I am no longer functioning as part of a team*” (12.3%); and (7) “*The EHR system is considered to be an extra load at work*” (9.7%) (31).

#### Barriers to EHRS implementation

**Figure 3:**
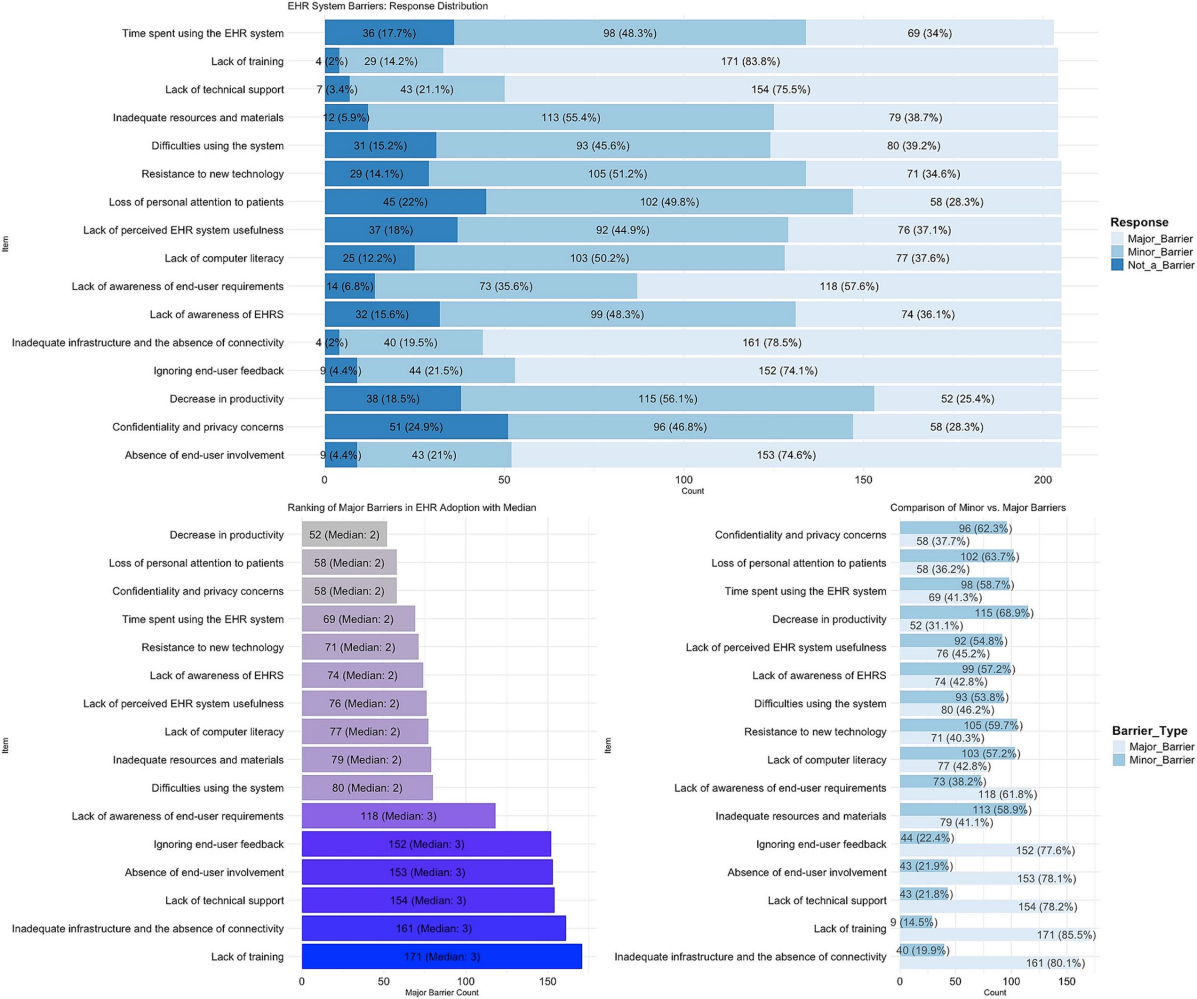
Barriers to implementation of EHRS in the PHCs.

As seen in Figure 3 certain elements are perceived as less inhibitory compared to others. The question that received the least amount of positive endorsement were: (1) “*Lack of training*” (83.8%); (2) “*Inadequate infrastructure and the absence of connectivity*” (78.5%); (3) “*Lack of technical support*” (75.5%) and; (4) “*The absence of end-user involvement in EHR implementation and software design*” (74.6%).

The items that were given the highest level of positive endorsement and reflected lesser barriers were: (13) “*The time spent using the EHR system*” (34%); (14) “*Confidentiality and privacy concerns*” (28.3%); (15) “*Concerns about loss of personal attention given to patients as entering patient information into the computer*” (28.3%) and; (16) “*Concern about a decrease in productivity during the use of the EHR system*” (25.4%). As seen in the below table, all listed items in the barrier scale were considered to be major or minor barriers (31).

### Inferential statistics

A series of non-parametric tests were conducted to examine the association between demographic variables and both negative attitudes toward EHRS and perceived levels of barriers to its implementation. The Kruskal-Wallis test was employed for occupation, region, experience in position, experience with EHRS, experience in using a personal computer, and age, revealing no statistically significant differences across these demographic groups. Specifically, for negative attitudes, *p*-values ranged from 0.166 (occupation) to 0.804 (experience in using a personal computer), while for perceived barriers, the highest p-value was observed for occupation (0.964) and the lowest for experience in using a personal computer (0.218). Similarly, the Mann–Whitney U test was used to assess differences in negative attitudes and perceived barriers based on gender, yielding *p*-values of 0.113 and 0.338, respectively, indicating no significant gender-based variations. These findings suggest that none of the examined demographic variables had a statistically significant impact on participants’ attitudes toward EHRS or their perceived level of barriers to its implementation (see Table 4).

The association between the key scales was determined using a Spearman’s correlation test. The association analysis revealed a significant negative correlation between impediments and a negative attitude toward the installation of EHRS (see Table 5). Nevertheless, all notable relationships are regarded as feeble. However, neither the Mann–Whitney U test nor the Kruskal Wallis test identified any significant differences between the groups.

## Discussion

Only 150 out of 2,259 PHCs have successfully implemented an EHRS, yet the project ultimately failed due to multiple challenges. This study identifies key barriers to EHRS implementation based on prior and ongoing experiences of the Saudi Ministry of Health. Data were collected through questionnaires and semi-structured interviews with both project implementation teams and EHRS end-users.

The findings indicate that the number of barriers outweighs the facilitators, with major obstacles including project scale, individual resistance to change, insufficient training and technical support, limited system compatibility, geographical constraints, software selection issues, and inadequate user engagement. Although PHC requirements were expected to facilitate implementation, this study highlights that large-scale EHRS deployments pose significant challenges. To mitigate these issues, phased implementation across multiple locations and engagement with multiple vendors to ensure system compatibility is recommended.

A critical barrier identified was the shortage of Health Informatics (HI) and IT specialists, a challenge observed in both developed and emerging nations (3, 10). This study strongly advocates recruiting qualified professionals externally to enhance EHRS implementation readiness. Additionally, high staff turnover, particularly among project executives and key decision-makers, disrupted continuity and contributed to project setbacks. While centralized management (CM) was beneficial for EHRS deployment, leadership transitions often led to policy and strategy changes, delaying implementation (33).

Training and technical support were significant concerns, with 83.8% of end-users citing inadequate training and 75.5% reporting insufficient technical support. These findings align with prior research, which demonstrated inconsistent satisfaction levels with training in Saudi Arabia’s secondary care settings (34–36). Notably, lack of technical support deficiencies were directly correlated with EHRS failure, reinforcing the necessity of robust support mechanisms during implementation (35, 36).

Geographical barriers, including inadequate infrastructure and connectivity issues, were major impediments, particularly in remote areas. These findings align with previous studies that highlight the risks of infrastructural challenges in EHRS projects, especially in developing countries (3, 37–39). Addressing these obstacles requires collaboration with telecom providers to ensure reliable connectivity and mitigate technological disparities (26, 39–42).

Another key technological challenge was the lack of EHRS interoperability, exacerbated by multiple vendors involved in the project. Prior research emphasizes that interoperability should be a priority during software selection, readiness assessment, and implementation planning (7, 16, 24, 36, 39–41, 43, 44). Standardizing software selection criteria and issuing a Request for Proposal (RFP) during pre-implementation can enhance system compatibility (45, 46). Furthermore, commercial EHRS solutions offer integration flexibility, which can support seamless interoperability (26).

Finally, the study highlights structural and procedural differences between PHCs in Saudi Arabia and those in other countries, complicating global system selection. Most international EHRS are designed to align with existing workflows, but in cases where local processes differ significantly, either system adaptation or custom development may be required. Addressing these workflow challenges is essential for successful EHRS adoption in Saudi PHCs.

In summary, EHRS implementation in Saudi PHCs faced significant hurdles, including project complexity, insufficient training and support, workforce limitations, geographical challenges, and interoperability concerns. Addressing these issues requires strategic planning, phased implementation, standardized software selection, infrastructure development, and enhanced workforce capacity to improve EHRS adoption and sustainability.

## Implications for EHRS implementation

The findings of this study have important implications for the successful adoption and scalability of EHRS, particularly in PHCs. To enhance adoption rates, future implementation efforts should focus on improving system usability, ensuring interoperability, and strengthening IT infrastructure, especially in geographically dispersed healthcare facilities. Additionally, fostering greater stakeholder engagement including collaboration with telecom providers, software developers, healthcare professionals, and policymakers can help address persistent challenges related to connectivity, data standardization, and technical support. A user-centered approach in system design and implementation is essential to overcoming end-user resistance and ensuring long-term sustainability.

While this study provides valuable insights, further research is needed to assess the long-term impact of EHRS on clinical workflow, patient outcomes, and cost-effectiveness. Future studies should also explore user experiences across diverse healthcare settings, evaluating how system usability, training, and support models influence EHRS adoption among different professional groups. Additionally, comparative studies of implementation models could provide deeper insights into effective strategies for EHRS deployment in varying healthcare environments.

## Limitations of the study

This study has several limitations that should be acknowledged. One key limitation is that the data collection tool did not assess the availability of specialized professionals, such as health informatics experts and IT specialists, who are critical in facilitating EHRS deployment. Future research should incorporate measures to evaluate organizational readiness, particularly in terms of workforce capacity, digital health competencies, and technical support resources, to provide a more comprehensive understanding of the human resource challenges impacting EHRS adoption.

Additionally, this study did not differentiate between rural and urban PHCs, which is an essential factor in understanding variations in infrastructure, internet access, and workforce availability. Identifying rural and urban PHCs was challenging due to inconsistent classification within healthcare records. However, given the potential benefits of EHRS, including telemedicine and enhanced healthcare access, future research should address this limitation by systematically exploring urban and rural disparities in EHRS adoption. Rural healthcare facilities often face greater resource constraints and digital health disparities, necessitating tailored implementation strategies to ensure equitable access to EHRS-enabled healthcare services. Examining how EHRS adoption influences healthcare accessibility in rural versus urban settings would provide valuable insights for policymakers and healthcare providers.

Furthermore, this study relied on self-reported data from qualitative interviews and online surveys, which are subject to response and recall biases. Participants may have over- or underreported certain challenges, influencing the interpretation of findings. Incorporating observational methods, usability testing, or direct workflow analysis in future research could provide a more accurate representation of real-world EHRS adoption and user interactions. Lastly, the generalisability of findings may be limited, as this study focused on a specific healthcare setting in Saudi Arabia. While the results may be relevant to other developing countries, they may not fully apply to regions with different regulatory frameworks, healthcare policies, or technological infrastructures. Future research should include cross-country comparisons to evaluate how policy environments and healthcare system structures influence EHRS adoption in diverse settings.

### Future research

Building on these findings, future research should focus on assessing the role of health informatics professionals and IT specialists in supporting EHRS deployment and ensuring workforce readiness. Comparative studies between rural and urban PHCs could provide deeper insights into geographical disparities in EHRS adoption and infrastructure limitations. Additionally, real-world usability assessments including workflow observations and system performance evaluations could enhance understanding of EHRS functionality and user experience. Further studies should also examine the long-term impact of EHRS on clinical efficiency, patient care outcomes, and cost-effectiveness, providing quantifiable evidence on healthcare improvements post-implementation. Research on the effectiveness of different training and support models across professional groups could help optimize future EHRS implementation strategies. Expanding research across various healthcare systems and countries will allow for comparative insights into best practices, policy interventions, and technological advancements that can inform more effective, scalable, and sustainable EHRS deployment globally.

By addressing these research gaps, policymakers, healthcare administrators, and system developers can make more informed, data-driven decisions, ultimately facilitating the successful and widespread adoption of EHRS. A strategic, evidence-based approach that considers workforce preparedness, geographical disparities, and real-world usability challenges will be key to ensuring long-term success in EHRS initiatives.

## Conclusion

This study contributes to the expanding body of research on EHRS implementation by identifying a comprehensive set of barriers affecting large-scale deployment in PHCs. The findings offer valuable insights for policymakers, healthcare administrators, and EHRS implementation teams, providing evidence-based strategies to enhance adoption and ensure long-term system sustainability. A key recommendation is the allocation of sufficient funding to support software selection, infrastructure development, and ongoing technical assistance, all of which are critical for ensuring a seamless and effective EHRS transition. A primary challenge identified was the lack of adequate training and support, emphasizing the need for comprehensive, role-specific training programs tailored to different user groups within PHCs. In contrast, confidentiality and privacy concerns were found to be less significant barriers, suggesting that resistance to EHRS adoption is primarily linked to technical and operational challenges rather than security apprehensions. Furthermore, the study found that demographic characteristics aside from participants’ occupation had minimal influence on end-user satisfaction, highlighting the importance of job-specific engagement strategies rather than broad demographic-based interventions.

The study also assessed the lessons learned from previous EHRS implementation efforts, revealing that the failure of earlier initiatives was predominantly due to insufficient connectivity, inadequate technical support, and high turnover in key leadership positions within the Saudi Ministry of Health. These challenges underscore the necessity for long-term policy planning, robust IT infrastructure, and stable leadership to facilitate a successful and sustainable digital transformation in PHCs.

## Data Availability

The raw data supporting the conclusions of this article will be made available by the authors, without undue reservation.
